# Iron overload resulting from the chronic oral administration of ferric citrate induces parkinsonism phenotypes in middle-aged mice

**DOI:** 10.18632/aging.102433

**Published:** 2019-11-07

**Authors:** Chao Huang, Wenjing Ma, Qihui Luo, Liangqin Shi, Yu Xia, Chengjie Lao, Wentao Liu, Yuanfeng Zou, Anchun Cheng, Riyi Shi, Zhengli Chen

**Affiliations:** 1 Laboratory of Experimental Animal Disease Model, College of Veterinary Medicine, Sichuan Agricultural University, Chengdu 611130, P.R. China; 2 Key Laboratory of Animal Disease and Human Health of Sichuan Province, College of Veterinary Medicine, Sichuan Agricultural University, Chengdu 611130, P.R. China; 3 Natural Medicine Research Center, College of Veterinary Medicine, Sichuan Agricultural University, Chengdu 611130, P.R. China; 4 Department of Basic Medical Sciences, College of Veterinary Medicine, Purdue University, West Lafayette, IN 47906, USA; 5 Sichuan Institute for Food and Drug Control, Chengdu 611130, P.R. China

**Keywords:** iron, ferric citrate, oxidative stress, neurodegeneration, parkinson’s disease

## Abstract

Iron homeostasis is critical for maintaining normal brain physiological functions, and its mis-regulation can cause neurotoxicity and play a part in the development of many neurodegenerative disorders. The high incidence of iron deficiency makes iron supplementation a trend, and ferric citrate is a commonly used iron supplement. In this study, we found that the chronic oral administration of ferric citrate (2.5 mg/day and 10 mg/day) for 16 weeks selectively induced iron accumulation in the corpus striatum (CPu), substantia nigra (SN) and hippocampus, which typically caused parkinsonism phenotypes in middle-aged mice. Histopathological analysis showed that apoptosis- and oxidative stress-mediated neurodegeneration and dopaminergic neuronal loss occurred in the brain, and behavioral tests showed that defects in the locomotor and cognitive functions of these mice developed. Our research provides a new perspective for ferric citrate as a food additive or in clinical applications and suggests a new potential approach to develop animal models for Parkinson’s disease (PD).

## INTRODUCTION

The challenges presented by neurodegenerative diseases (NDs) in an aging population make research into the pathogenesis of these diseases urgently needed [1]. Brain iron abnormalities have been implicated in various NDs, including Alzheimer’s disease (AD), Huntington’s disease (HD), amyotrophic lateral sclerosis (ALS), multiple sclerosis (MS) and especially in Parkinson’s disease (PD) [2, 3]. With postmortem, MRI and transcranial ultrasound, the excessive iron deposition is consistently demonstrated in the substantia nigra and basal ganglia of the brain in PD patients, and a 25% to 100% increase of the iron levels in substantia nigra is present according to the quantitative data [4, 5]. Iron plays important roles in multiple biochemical processes by facilitating two-way electron transport, and it functions as a critical cofactor of many proteins involved in cellular proliferation, differentiation, and apoptosis [6, 7]. Given that the metabolic activity of brain is high and the iron functions as an enzymatic cofactor in myelinogenesis, the concentration of iron in the brain is high [8]. Disorders of iron metabolism, both iron deficiency and iron overload, could be harmful to the brain and a cause of neurological diseases. The lack of iron results in the construction of abnormal neural connections or the abnormal synthesis of neurotransmitters synthesis, and it is implicated in a range of neurological disorders primarily clinically characterized by cognitive, physical and social impairments, such as restless leg syndrome and cognitive dysfunctions [9–11]. On the other hand, as the redox reactivity of iron is high but not selective, iron overload in the brain will disrupt redox balance and drive oxidative stress, which is widely associated with NDs [12]. Cells with active iron metabolism are more sensitive to this iron toxicity, such as dopaminergic neurons that need iron for dopamine synthesis [13]. Therefore, the homeostasis of iron, which mainly depends on the balance between iron uptake and iron release, needs to be well controlled in the brain [14].

Iron is taken up through the blood-brain barrier (BBB) in the brain, from the basolateral membrane of endothelial cells to the cerebral compartment. The present evidence suggests that the transferrin/transferrin receptor/divalent metal transporter 1 (Tf/TfR/DMT1) pathway is the major pathway for iron transport across the BBB, which includes the processes of binding, endocytosis, acidification, dissociation and translocation [15, 16]. On the other hand, brain iron release is dependent on the only iron exporter currently identified, ferroportin-1 (Fpn1), which releases iron into circulation to be loaded onto Tf by collaborating with ceruloplasmin or ferroxidase [17, 18]. Although more than two-thirds of the total amount of iron needed in the body is from the degradation of senescent red blood cells and the rest comes from the diet [19], according to the WHO, iron deficiency is the most common nutritional disorder in the world, especially in developing countries [20, 21]. In addition, iron deficiency is a multifactorial condition in which the incidence increases with age in adulthood, and a substantially higher prevalence is present in middle-aged and elderly populations than in young populations [22, 23]. Thus, rational iron supplementation is important to maintain iron homeostasis in the body and, of course, in the brain. Many different types of iron supplements are available on the market, including ferrous and ferric iron salts, such as ferrous sulfate, ferrous gluconate, ferric citrate, and ferric sulfate [24]. Therefore, as trace element supplementation becomes increasingly normalized, additional attention must be paid to the side effects of excessive iron supplementation.

The toxicity of iron overload on brain functions was widely studied in iron injection models, and the intranigral infusion of ferric citrate or some other iron carriers resulted in increased sensitivity to 1-methyl-4-phenyl-1,2,3,6-tetrahydropyridine (MPTP), enhanced oxidative stress in nigral neurons, and accelerated dopamine (DA) depletion [25, 26]. However, the toxicity of overloaded iron intake by oral supplementation on brain functions has rarely been explored. A study performed by Sobotka et al. found increased brain iron concertation and some neurobehavioral dysfunctions in rats with dietary iron overload [27], while Schroder et al. reported memory deficits in adult rats orally administered excessive ferromyn, a common iron supplement [28]. Ferric citrate is another common oral iron supplement and is widely used as a food additive in flour, formula milk, crackers, etc. Ferric citrate is on the registered list of food ingredients from the Ministry of Health, Labour and Welfare of Japan, and the Code of Federal Regulations (CFR) of the US [29]. No evidence for chronic toxicity or tumorigenicity of ferric citrate was found in mice administered long-term and low-dose (0.06% and 0.12%) supplementation [30], and no changes in the brain weight of adult rats were observed under high-dose ferric citrate (up to 4%) oral supplementation for 13 weeks [31]. However, it was reported that the oral administration of high-dose ferric citrate quickly induced a significant increase in iron in the male rat brain [32]. Therefore, it is reasonable to suspect that oral supplementation with high-dose ferric citrate would be harmful to the structure or function of the brain, especially under long-term conditions in middle-aged or elderly subjects, who are more sensitive to iron overload and its resulting oxidative stress [33, 34]. In this study, we aimed to address this issue and investigate the effects of the chronic oral administration of ferric citrate on brain histology and neurobehavioral functions in middle-aged mice to provide new perspectives for iron supplementation.

## RESULTS

### Chronic oral administration of ferric citrate induces selective iron overload in the brain

**Figure 1:**
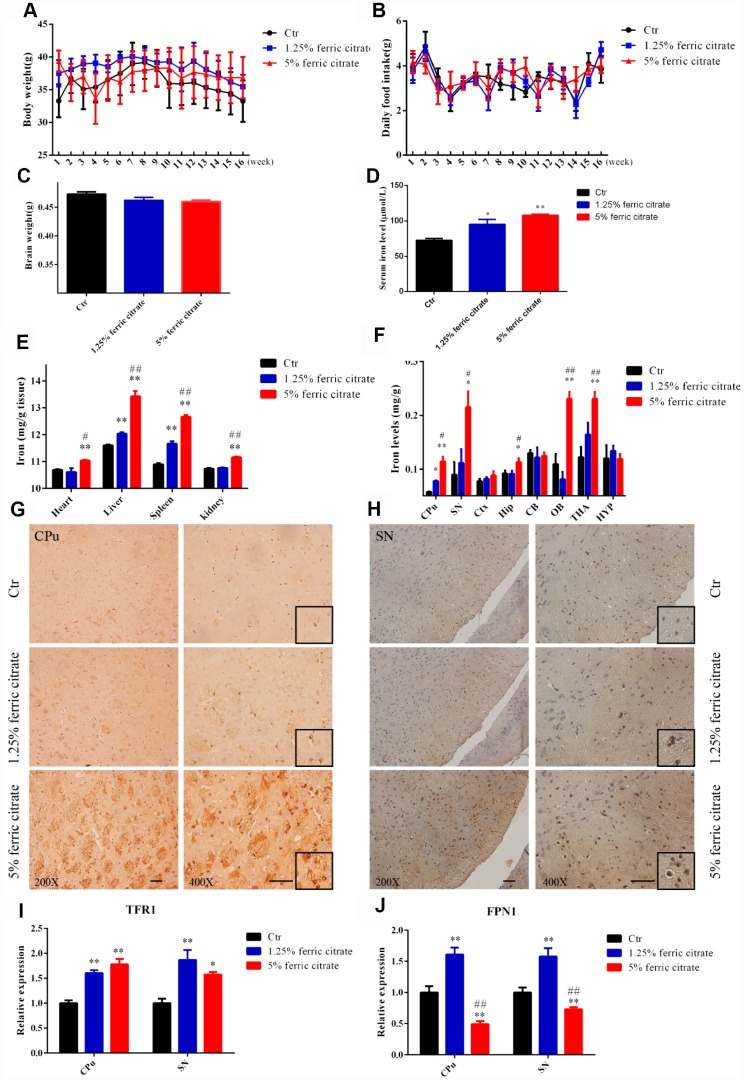
**Chronic oral administration of ferric citrate induces selective iron overload in the brain.** (**A** to **C**) Quantifications show no differences in the body weight, daily food intake and brain weight of mice supplemented with ferric citrate. Error bars indicate SD. (**D**) Quantification shows the increased serum iron levels of mice supplemented with ferric citrate (N=10). Error bars indicate SEM. (**E**) Quantification shows the increased peripheral tissue iron levels of mice supplemented with ferric citrate (N=10). Error bars indicate SEM. (**F**) Quantification shows the selective iron overload in the brains of mice supplemented with ferric citrate (N=10). Error bars indicate SEM. (**G** and **H**) Representative images from Prussian blue staining show the excessive iron accumulation in the Cpu and SN of mice supplemented with ferric citrate. Bars, 100 μm. (**I**) qRT-PCR shows the increased mRNA levels of TFR1 in the Cpu and SN of mice supplemented with ferric citrate (N=5). Error bars indicate SEM. (**J**) qRT-PCR shows that the mRNA levels of FPN1 increased in the Cpu and SN of mice from the 1.25% ferric citrate group but decreased in those of mice from the 5% ferric citrate group (N=5). Error bars indicate SEM. Compared with the Ctr group, *p<0.05 and **p<0.01. Compared with the 1.25% ferric citrate group, ^#^p<0.05 and ^##^p<0.01.

To evaluate the effects of the chronic supplementation of ferric citrate on the brain functions of middle-aged subjects, 9-month C57BL/6 mice were intragastrically administered ferric citrate (2.5 mg or 10 mg) daily for 16 weeks. Weekly body weight and food intake, as well as brain weight, were measured, and no significant differences among the different groups were observed during the experimental period (Figure 1A–1C). The accumulation of iron in the body was analyzed after the mice were killed. The absorption of ferric citrate led to a robust increase in the serum iron level in the ferric citrate groups (Figure 1D), and the accumulation of iron was also observed in the heart, liver, spleen and kidney, especially in the 5% ferric citrate group (Figure 1E). In the brain, the iron level was quantified by flame atomic absorption analysis. We found that the accumulation of iron was dramatically increased in the substantia nigra (SN), caudate putamen (CPu), olfactory bulb (OB) and thalamus (THA) after ferric citrate administration, and the hippocampal (Hip) iron level moderately increased in the high-dose ferric citrate group, but no such changes were detected in the cortex (Ctx), cerebellum (CB) and hypothalamus (HYP) (Figure 1F). The accumulation of iron in the SN and CPu, further confirmed by Prussian blue staining, indicated that there was a marked dose-dependent increase in the positive signals in the ferric citrate groups (Figure 1G and 1H). Increased iron transport, as indicated by the upregulated expression of the major iron uptake transporter TFR1, may be responsible for the accumulation of iron in the brain after ferric citrate supplementation (Figure 1I). Excessive iron is excreted by the protective exporter mechanism of the brain, and FPN1 functions as an iron efflux transporter in the brain [35]. A robust increase in FPN1 expression was detected in the 1.25% ferric citrate group, while a dramatic decrease was observed in the 5% ferric citrate group (Figure 1J), suggesting the dose- and time-dependent destruction of the balance between iron uptake and export with ferric citrate supplementation. These data demonstrated that the chronic oral supplementation of ferric citrate, especially at a high dose, could lead to an accumulation of iron in the brain with selective regional differences. This finding is consistent with previous reports that the concentration of iron varies greatly among different regions of the brain, and more iron tends to accumulate in the regions associated with motor functions than nonmotor-related regions [36, 37].

### Motor and cognitive defects are associated with iron accumulation in ferric citrate-supplemented mice

**Figure 2:**
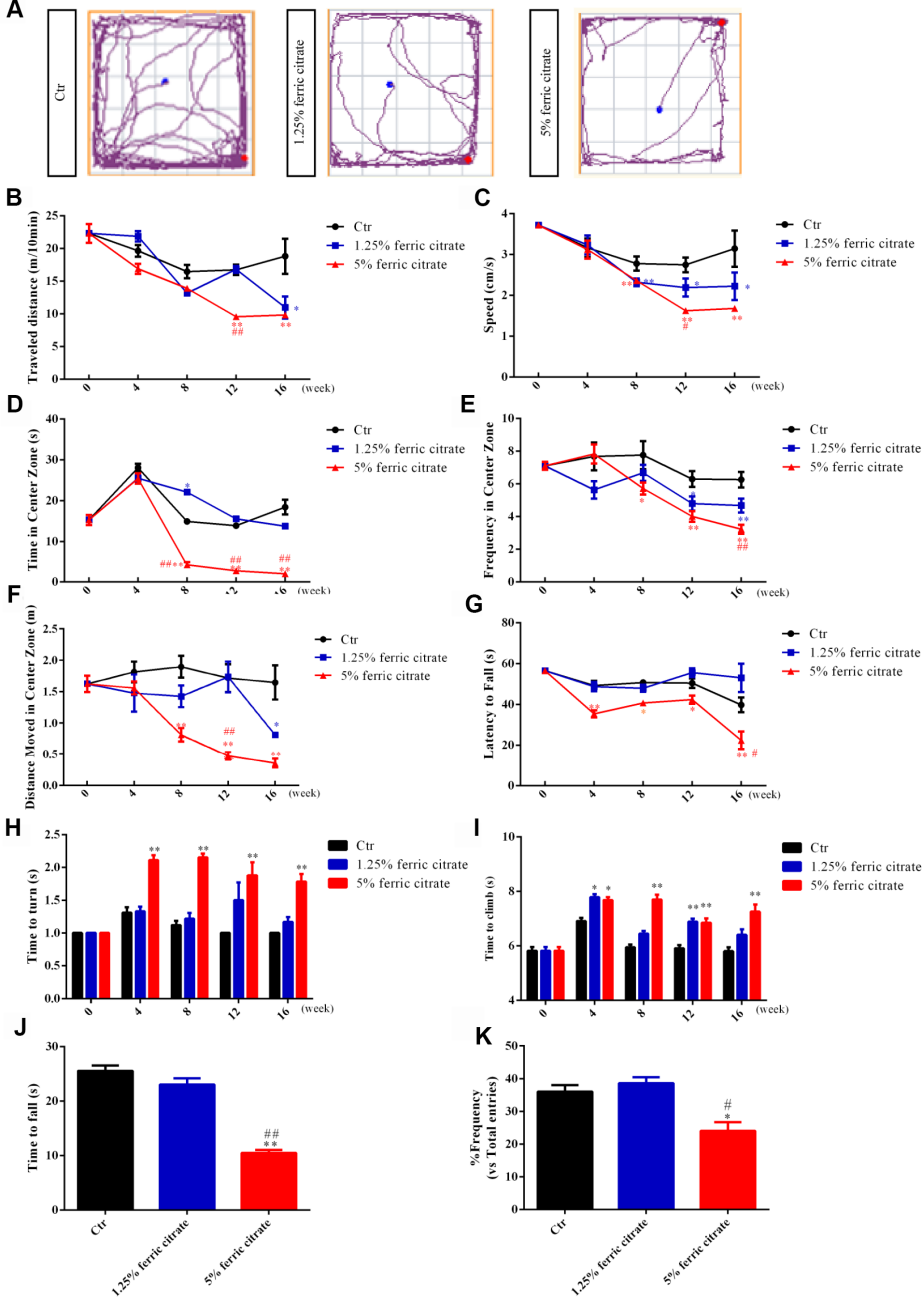
**Motor and cognitive defects are associated with iron accumulation in ferric citrate-supplemented mice.** (**A**) Representative maps of mouse activities in the open field test. (**B**) Effect of ferric citrate on the distance traveled by mice. (**C**) Effect of ferric citrate on the speed of mice. (**D**) Effect of ferric citrate on the time mice spent in the center zone. (**E**) Effect of ferric citrate on the frequency mice moved into the center zone. (**F**) Effect of ferric citrate on the distance mice traveled in the center zone. (**G**) Effect of ferric citrate on the fall latency of mice. (**H** and **I**) Effect of ferric citrate on the performance of mice in the pole test. (**J**) Effect of ferric citrate on the time to fall of mice in the traction test. (**K**) Effect of ferric citrate on the cognitive functions of mice, as evidenced by the quantification of their frequency to enter the novel arm in the Y-maze test. Error bars indicate SD. Compared with the Ctr group, *p<0.05 and **p<0.01. Compared with the 1.25% ferric citrate group ^#^p<0.05 and ^##^p<0.01.

Increasing evidence has demonstrated that excessive iron accumulation in selective brain regions may induce oxidative stress-related damage and thereby cause neurobehavioral dysfunctions that are widely implicated in NDs [38, 39]. Considering the potential accumulation of iron in the brain after ferric citrate supplementation, multiple behavioral tests were performed during the experiment. First, locomotor functions were assessed by an open field test. Representative maps of mouse activities showed that the oral administration of ferric citrate could reduce the mobility of mice (Figure 2A). Further statistical results found that the total travel distance and the speed, frequency, distance and time spent in the center zone were decreased in the ferric citrate groups in a time- and dose-dependent manner (Figure 2B–2F). Second, the accelerated rotarod test and pole test were performed to measure the gross motor skill and motor coordination of these mice [40, 41]. Quantification showed that compared with the mice in the other groups, the mice supplemented with 5% ferric citrate displayed a significant time-related decrease in fall latency (Figure 2G), while the times required for the mice to turn around and descend to the floor in the pole test were remarkably increased (Figure 2H and 2I). Then, in the last experimental week, the grip strength of these mice was measured with a traction test [42], and the results showed that the mice from the 5% ferric citrate supplementation group spent much less time on the rope than those from the other two groups (Figure 2J). In addition, as mentioned above, the iron concentration in the hippocampus was also increased in the 5% ferric citrate-supplemented mice; thus, we also performed a Y-maze test to assess the cognitive function of these mice [43]. As shown in Figure 2K, the frequency that mice entered the novel arm of the Y-maze was lower in the 5% ferric citrate group than in the control group. These results showed the effects of the chronic oral intake of ferric citrate on impairing the motor and cognitive functions of middle-aged mice, and these behavioral defects are known to be indicative of experimental parkinsonism [44]. Therefore, we consider these middle-aged ferric citrate over-supplemented mice to be a potential PD animal model, which will be a powerful tool for research on PD mechanisms and drugs.

### Iron overload induced by ferric citrate supplementation causes neurotoxicity in SN and CPu

**Figure 3:**
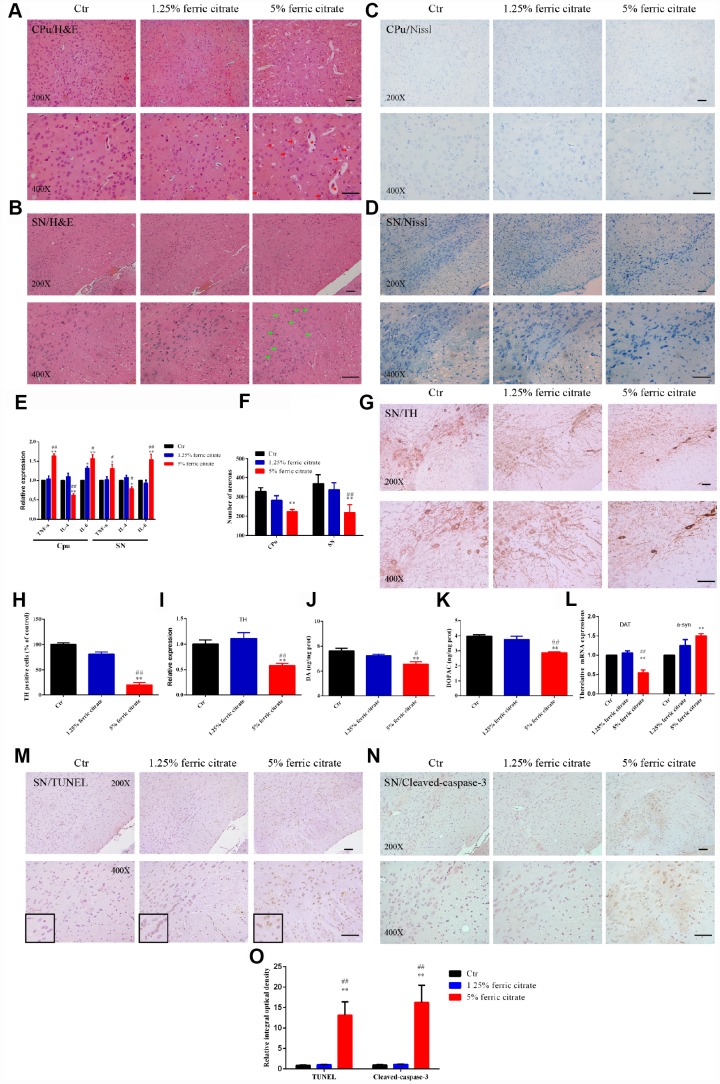
**Iron overload induced by ferric citrate supplementation causes neurotoxicity in the SN and CPu.** (**A** and **B**) Representative images of H&E staining display the histopathological damage in the CPu and SN induced by ferric citrate supplementation. Red arrows show white matter edema, red stars show vasodilatation, and green arrows display nerve cell swelling. (**C**, **D** and **F**) Representative images and quantification of NISSL staining display the decreased numbers of neurons in ferric citrate-supplemented mice. Error bars indicate SD. (**E**) qRT-PCR showed increased mRNA levels of TNF-α and IL-6 and decreased expression of IL-4 in the Cpu and SN of mice supplemented with ferric citrate (N=5). Error bars indicate SEM. (**G** and **H**) Representative images and quantification of TH staining display decreased numbers of dopaminergic neurons in the SN of mice supplemented with ferric citrate. Error bars indicate SD. (**I**) qRT-PCR shows decreased mRNA levels of TH in the SN of mice supplemented with ferric citrate (N=5). Error bars indicate SEM. (**J** and **K**) Quantifications show the decreased levels of DA and DOPAC in mice supplemented with ferric citrate. Error bars indicate SEM. (**L**) qRT-PCR show the mRNA levels of DAT and a-syn in the SN of mice supplemented with ferric citrate (N=5). Error bars indicate SEM. (**M** to **O**) Representative images and quantification from TUNEL and cleaved caspase-3 staining display the increased neuronal apoptosis in the SN of mice supplemented with ferric citrate. Bars, 100 μm. Compared with the Ctr group, *p<0.05 and **p<0.01. Compared with the 1.25% ferric citrate group, ^#^p<0.05 and ^##^p<0.01.

Given that the brain iron accumulation resulting from the chronic oral uptake of ferric citrate caused motional and cognitive defects, we further explored the underlying histopathological damage. As shown by H&E staining, nerve cell swelling was present in the SN, while white matter edema and vasodilatation were observed in the CPu of the mice supplemented with 5% ferric citrate (Figure 3A and 3B), but no observable pathological changes were found in the 1.25% ferric citrate and control groups. Moreover, cell swelling or white matter edema was also found in the globus pallidus, thalamic and red nuclei (Supplementary Figure 1). These histopathological findings suggested the occurrence of neuroinflammation after ferric citrate supplementation, which was further evidenced by detecting the expression of inflammatory factors. As shown in Figure 3E, the expression levels of the proinflammatory factors TNF-α and IL-6 were increased, while the expression of the anti-inflammatory factor IL-4 was suppressed in the 5% ferric citrate group (Figure 3E). Nissl staining was performed to quantify the numbers of neurons in the SN and CPu and displayed a remarkable neuronal loss in the 5% ferric citrate group (Figure 3C, 3D and 3F). Specifically, the neurons lost in the SN were dopaminergic neurons, as indicated by tyrosine hydroxylase (TH) staining and qRT-PCR (Figure 3G–3I), which further resulted in the depletion of dopamine (DA) and its metabolite (dihydroxyphenylacetic acid, DOPAC) in the CPu (Figure 3J and 3K). Besides, the expression of dopamine transporter (DAT) in striatum was also reduced in 5% ferric citrate group (Figure 3L). Moreover, our study further demonstrated that cellular apoptosis was responsible for the neuronal loss in the SN and CPu, as many more positive signals were observed in the subjects in the 5% ferric citrate group by TUNEL and cleaved caspase-3 staining (Figure 3M to 3O). Dopaminergic neurons constitute a major source of dopamine, which is one of the most important neurotransmitters involved in the nigrostriatal pathway that controls voluntary motor movement [45]. Therefore, the neurotoxicity to SN dopaminergic neurons after the chronic oral uptake of ferric citrate may be the cause for the behavioral defects previously observed. Lewy bodies are important clinical manifestation in PD patients, but in our model, even we have detected increased expression of alpha synuclein (a-syn) in the CPu from 5% ferric citrate group (Figure 3L), but we didn’t observe any Lewy body both in SN and CPu (Data not shown).

### Oxidative stress-induced neuronal loss is implicated in the neurotoxicity of ferric citrate supplementation

**Figure 4:**
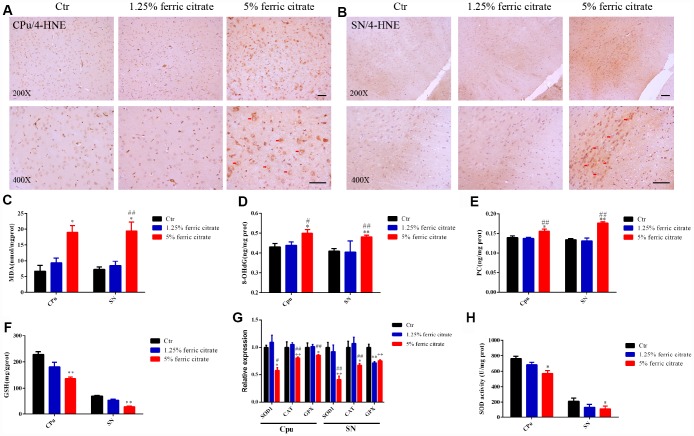
**Oxidative stress-induced neuronal loss is implicated in the neurotoxicity of ferric citrate supplementation.** (**A** and **B**) Representative images of immunohistochemical 4-HNE staining show the accumulation of lipid peroxidation in the CPu and SN induced by ferric citrate supplementation. (**C**–**E**) Quantifications show the increased peroxidation of lipids, DNAs and proteins in the CPu and SN induced by ferric citrate supplementation. (**F**) Quantification shows the decreased GSH levels in the CPu and SN induced by ferric citrate supplementation. (**G**) qRT-PCR shows the decreased mRNA levels of typical antioxidant genes in the CPu and SN of mice supplemented with ferric citrate (N=5). (**H**) Quantification shows the decreased activities of SOD in the CPu and SN induced by ferric citrate supplementation. Error bars indicate SEM. Bars, 100 μm. Compared with the Ctr group, *p<0.05 and **p<0.01. Compared with the 1.25% ferric citrate group, ^#^p<0.05 and ^##^p<0.01.

As a transition metal, iron is capable of generating hydroxyl radicals via the Fenton reaction. Consequently, elevated iron deposition induces oxidative stress and triggers the accumulation of oxidative damage and neuronal death, which is widely implicated in NDs [8, 46]. To explore whether oxidative stress was induced by chronic ferric citrate supplementation, oxidative damage was analyzed in the SN and CPu of mice. Lipid peroxidation was evaluated by 4-hydroxynonenal (4-HNE) staining, and a widespread increase in 4-HNE positive signals was observed in the SN and CPu of mice, especially in the 5% ferric citrate group (Figure 4A and 4B). This increased 4-HNE level was accompanied by an increase in malondialdehyde (MDA) (Figure 4C), another product generated from lipid peroxidation [47]. Oxidative damage to proteins and DNAs was quantified by a protein carbonylation assay kit and an 8-hydroxydeoxyguanosine (8-OHdG) assay kit, respectively [48]. The data showed that markedly higher levels of protein carbonylation (PC) and 8-OHdG were present in the SN and CPu of mice in the 5% ferric citrate supplementation group than in the control group (Figure 4D and 3E). Iron accumulation was reported to result in the depletion of reduced glutathione (GSH), resulting in decreased oxidative defense [49]. Consistent with this observation, we detected a significant decrease in GSH in the SN and CPu of mice from the 5% ferric citrate group (Figure 4F). In addition, the expression levels of multiple critical antioxidant defense genes, such as superoxide dismutase 1 (SOD1), catalase (CAT) and glutathione peroxidase (GPX), were downregulated in the SN and CPu of mice supplemented with 5% ferric citrate (Figure 4G), and the activities of SOD in these tissues were also reduced (Figure 4H). Accumulating oxidative damage triggered cellular apoptotic processes, as shown in Figure 3L and 3M. This finding suggested that the oxidative stress generated in the ferric citrate-supplemented mice was involved in dopaminergic neuronal loss and neurobehavioral defects.

## DISCUSSION

The potential neurodegenerative effects of iron overload in specific brain regions have been explored before. Correlations among iron accumulation, DA/DOPA concentrations, and progressive nigral atrophy have been found in models intranigral infused with different iron reagents, such as ferric chloride, ferric citrate and ferric ammonium citrate [25, 26, 50]. Iron overload models induced by oral supplementation were also preliminary studied by some groups. Sobotka et al. fed adult weanling rats diets composed of different doses of iron for 12 weeks, and reduced total activity, impaired avoidance learning and prepulse inhibition were detected in the high-dose group (20 000 ppm). However, the iron concentrations in different brain regions and the pathological injuries responsible for behavioral defects were not evaluated in that study [27]. Iron overloaded diets were administered to adult rats for 7 days by Yu et al., who found increased iron/MDA and decreased GSH in the brain [51]. The far-reaching effects of postnatal iron over-supplementation on learning behavior were also evaluated in rat pups. Short-term (3 days) administration of excessive ferromyn to 10-day-old rats resulted in significantly increased iron concentrations in the SN and memory defects at adult ages [28]. In this study, we first systematically evaluated the effects of long-term oral iron overload on neurobehavior and its underlying mechanism in middle-aged subjects. Selective iron deposition was observed in different brain regions, which is different from a previous study with short-time administration [51]. The iron accumulation induced oxidative stress in the SN/CPu and further induced neuronal apoptosis, which led to dopaminergic neuronal loss and defects in the motor and cognitive functions of the mice in our study. These data reveal the neurotoxicity of the chronic oral uptake of ferric citrate to the brain of middle-aged mice in a region-selective and time-dependent manner.

In addition to iron supplements, ferric citrate is also used as a phosphate binder to treat hyperphosphatemia both in patients with dialysis- and nondialysis-dependent chronic kidney disease (CKD) [52]. The typical initial dose of ferric citrate hydrate is approximately 500 mg 3 times per day after meals; then, the dosage is adjusted based on the concentration of serum phosphorus, and a maximum daily dose of 6 000 mg ferric citrate hydrate is suggested. Ferric citrate hydrate is composed of approximately 20% water by weight; thus, the maximum daily dose of ferric citrate was approximately 4800 mg [29]. In our study, the daily doses of ferric citrate were approximately 83.3 mg/kg and 333.3 mg/kg in the 1.25% and 5% groups, respectively. These daily doses could be converted to equivalent doses for human adults (subjects with 70 kg bodyweight) according to previously described [53, 54] of approximately 646.5 mg and 2585.9 mg. These equivalent doses (646.5 mg and 2585.9 mg) are less than the currently suggested maximum daily dose for ferric citrate (4800 mg). As progressive neurobehavioral dysfunctions and accumulating brain pathological damages were present in the mice administered ferric citrate in our study, we think that more attention needs to be directed to the current suggested dose of ferric citrate or ferric citrate hydrate both for the treatment of hyperphosphatemia and as an iron supplement, especially in cases with long-term medication and middle or even elderly ages.

Considerable injuries occur before the onset of clinical symptoms in PD patients, making the identification of early events a challenge. Animal disease models, both toxic and genetic, are important for the pathophysiological studies, new medical target identification, and risk factor screening of PD. MPTP injection is the most widely used method to generate PD models in mice and nonhuman primates [55, 56]. A profound loss of DA in the CPu/SN resulting from damage to the nigrostriatal DA pathway is present after MPTP injection [57]. However, an acute or subacute pathological process is displayed in MPTP models, so it is not suitable for the observation of developing pathological processes or screening early diagnostic indicators of PD. Such defects are also present in models induced by 6-hydroxydopamine (6-OHDA). Moreover, the establishment of this model is not very convenient because 6-OHDA cannot cross the blood-brain barrier, and it can only be directly injected into the SNc, medial forebrain bundle or striatum to induce parkinsonism [58]. Rotenone is another commonly used agent to develop PD models. In contrast to MPTP and 6-OHDA, the induction of parkinsonism by rotenone can be chronic and continuous [59]. Modeling time can last up to approximately 2 months, and many features of PD can be reproduced in this model [60]. However, the mortality of this model can be high, and the replication is poor [61]. In our study, we found that the chronic oral administration of ferric citrate could induce the phenotypes of parkinsonism in mice, including the selective degeneration of the dopaminergic neurons, iron accumulation and oxidative stress in the CPu and SN, as well as defects in locomotor and cognitive functions, which suggest that this model could be a potential animal disease model for PD. And, this model has two major advantages over existing ones. First, the longer modeling time and progressive behavioral and pathological development make this model more suitable for monitoring the early events and screening the early diagnostic indicators of PD. Second, selectively iron deposition in this model will make it valuable for the study of PD treatments. For example, the emergence of iron mismanagement has elicited interests in developing neurotherapeutic strategies with chelation therapies, which have been tested in cell models, animal models and clinical studies. Desferrioxamine (DFO), a cell impermeable iron chelator, has been reported to reduce DA neuronal degeneration both in the 6-OHDA-induced rat model and MPTP-treated mouse model [62]. While, VK28, a strong brain permeable iron chelator, also displays neuroprotection effect on the PD progression in the 6-OHDA-treated rat model [63]. Besides, neurorestorative effects of iron chelation on PD have even been reported in some studies [64–66]. However, iron deposition is not a typical feature for both MPTP models or 6-OHDA models, and this is the advantage of our model. Thus, this model could be a more suitable choose to evaluate the effects of iron chelator on PD, and to study whether this protection of iron chelator is dependent on chelation of iron or not. In addition to these advantages, limitations need to be thought for our model. First, considerations and studies of the impact of peripheral iron overload on the progression of PD in this model are needed. Second, as region specific iron may vary depending on different PD stages, the dosage of iron supplements during disease progression may be different and could be changed.

In conclusion, we first reported that long-term oral supplementation with high-dose ferric citrate to middle-aged mice caused selective iron accumulation in the SN/CPu, which further induced oxidative stress-mediated dopaminergic neuronal loss. The defects in locomotor and cognitive functions resulting from these histopathological injuries were observed in these mice. Our research provides a new perspective for ferric citrate in food additives and clinical applications and a new potential method for developing PD animal models.

## MATERIALS AND METHODS

### Animal care and maintenance

All animal works were performed in accordance with the requirements of “*The National Institutes of Health Guide for the Care and Use of Laboratory Animal*” and were approved by the Animal Welfare and Animal Ethics Committee of Sichuan Agricultural University, China. Sixty C57BL/6 male mice (9 months old) were obtained from Beijing Weitong Lihua Experimental Animal Technology Co. Ltd. and maintained in individual cages in a specific pathogen-free environment with an automatically controlled 12-hour light/dark cycle and free access to food and water for 7 days. Then, the mice were randomly divided into 3 groups, with 20 mice in each group, including the control group (Ctr), 1.25% ferric citrate group and 5% ferric citrate group. The dosages of ferric citrate refer to Toyoda’s study [31]. We used male mice to generate our model because the males do not have the physiological cycle of the females, and the hormones in males maintain a dynamic balance, which is important for the experimental stability and repeatability. Before ferric citrate administration, 5 g ferric citrate was dissolved in 100 ml physiological saline to obtain a 5% ferric citrate solution when heated to boiling. Then, a gradient dilution was performed to obtain a 1.25% ferric citrate solution. Under these conditions, the solubility of ferric citrate was good, and the solution was clear. In our study, 0.2 ml of ferric citrate solution was intragastrically administrated to the mice each day, and an equal volume of physiological saline was intragastrically administrated to the mice in the control group. Therefore, the daily chemical intake was 2.5 mg and 10 mg in the low- and high-dose ferric citrate groups, respectively (Table 1). The intragastric administration of ferric citrate was performed daily beginning at 10:00 am for 16 weeks, and the body weight and food intake were determined weekly. Behavioral tests were conducted to evaluate the effects of ferric citrate on locomotor and cognitive functions during the first week of the trial and each subsequent month. At the end of the trial, all mice from each group were randomly and evenly divided into two groups. Subjects from one group were killed by decapitation, and then the brain, heart, liver, spleen and kidney were obtained and frozen in liquid nitrogen for RNA extraction and biomedical assays. Subjects from the other group were anesthetized with 4% chloral hydrate and perfusion-fixed with 4% paraformaldehyde. The same organs were obtained and fixed in 4% paraformaldehyde and cryopreserved for subsequent histological and immunostaining.

**Table 1:** Animal treatments.

**Group**	**The volume of intragastric administration (ml/day)**	**Chemical intake (mg/day)**	Conversion dose* (mg/kg/day)
Ctr	0.2	0	0
1.25%	0.2	2.5	83.3
5%	0.2	10	333.3

Ctr, control group that was intragastrically administered physiological saline.

*The conversion dose of ferric citrate was calculated according to the initial average bodyweight of the mice (~30 g)

### Detection of iron

The levels of iron in serum, heart, liver, spleen and kidney were determined by a Colorimetric Assay Kit (Nanjing Built Biology, Nanjing, China) according to the manufacturer’s instructions. The iron levels in the brain were quantified by flame atomic absorption analysis. Briefly, the sample was digested in concentrated nitric acid at 180 °C for 2 h (Mars 6, Thermo Fisher Scientific Inc. Waltham, MA, USA). Then, the iron concentration was determined by flame atomic absorption spectrometry (Model PE-800, PerkinElmer, USA). Validation of the mineral analysis was conducted using green tea or bovine liver powder as a standard reference material (National Institute of Standards and Technology, Beijing, China).

### Perls staining

First, paraffin slices were dewaxed, soaked in distilled water for 3 min, and then incubated in Perls solution containing 7% potassium ferrocyanide and 3% hydrochloric acid at a 1:1 ratio for 30 min, followed by three washes in PBS. Second, the slices were soaked in 1% H_2_O_2_ for 30 min and washed 3 times with distilled water. Finally, the slides were incubated in PBS containing 0.25 mg/mL DAB and 0.02% H_2_O_2_ for 10 min, counterstained for 5 min, dehydrated with gradient alcohol and mounted with xylene.

### Behavioral studies

### Open field test

The open field test was performed according to a previous report [67]. An open field device was provided by Jiangsu Cyrus Biotechnology Co. Ltd. and consisted of a white square arena (50×50 cm^2^, 50 cm high) and video capture system. The test was initiated by placing the mouse at the center of the arena and allowing the mouse to explore the arena for 10 min. Then, locomotor activities were analyzed by using an ANY-maze animal behavior video analysis system (Global Biotech Inc., USA).

### Accelerated rotarod test

An accelerated rotarod test was performed according to a previous study [68]. An accelerated rotarod experimental device was provided by Jiangsu Cyrus Biotechnology Co. Ltd. The mice were placed in a uniform rotating rod (rotation speed 5 r/min) with a 9 cm wide lane and a 3 cm diameter rotating rod. When the speeds of the mice were stable, they underwent a uniform acceleration process (maximum time of 5 min, speed increases every 8 s) three times. The average retention time on the revolving rod was determined. The day before the test, all the mice were pretrained on the rotarod three times (1 h interval).

### Pole test

The pole test was performed as previously described [41]. Briefly, mice were placed vertically on a 50 cm tall pole with a 1 cm diameter, after which the mice make a 180° turn and return to the base of the pole. The day before the test, the mice were habituated to the pole 5 times. During the test, the amount of time was recorded for the mouse to turn toward the ground (time to turn) and to reach the ground (time to climb). Each mouse underwent five trials, and the average times were quantified.

### Traction test

Both forelimbs of the mice were hung on a wire with a diameter of 1.5 mm, 30 cm above the ground, and a cap was placed 1 cm above the rod to prevent the mice from turning over. The time before landing was recorded, and each test interval was 1 min. A total of 5 tests were conducted to average.

### Y-maze test

A food reward type of Y-maze test was performed as previously described [69]. Briefly, all mice were subjected to a 2 Y-maze test trials separated by a 1-h intertrial interval to assess spatial recognition memory. The mice were fasted the day before the test. The first stage was the training period. The new arm was blocked by the partition, and the mouse was placed in the labyrinth and allowed to freely move from the starting arm for 10 min. The second stage was the detection period. Food was placed at the new arm, and the mouse was allowed to freely move in the maze for 5 min. Data are expressed as the percentage of novel arm entries made during the 5-min trial.

### Histopathologic analysis

### H&E and Nissl staining

Organs were fixed in 4% paraformaldehyde before paraffin sectioning. Then, hematoxylin and eosin (H&E) and Nissl staining were performed according to the instructions provided by the manufacturer (Beyotime, Shanghai, China). The number of neurons was quantified by Image Pro Plus (MEDIA CYBERNETICS, USA).

### Immunohistochemical staining

Organs were fixed in 4% paraformaldehyde before paraffin sectioning. Then, the paraffin slices were dewaxed and subjected to immunohistochemical staining with standard methods [70] and primary antibodies, including tyrosine hydroxylase (TH) (ENZO, USA, 1:1000), 4-hydroxynonenal (4-HNE) (Abcam, USA, 1:1000) and cleaved caspase-3 (ZEN BIO, China, 1:100). Positive signals were visualized using colorimetric detection with diaminobenzidine (DAB), and the hematoxylin indicated the nucleus. Finally, the images were photographed with a microscope (BX63, Olympus).

### Enzyme-Linked Immunosorbent (ELISA) and Biochemical Reaction assay

The levels of dopamine (DA), 3,4-dihydroxyphenylacetic acid (DOPAC), 8-hydroxy-2- deoxyguanosine (8-OHdG) and protein carbonylation (PC) were measured by ELISA assay kits (Shanghai Enzyme Linked Organisms, Shanghai, China) according to the manufacturer’s instructions. The activity of superoxide dismutase (SOD) and the levels of glutathione (GSH) and malondialdehyde (MDA) were determined by a biochemical reaction assay kit (Nanjing Built Biology, Nanjing, China) according to the manufacturer’s instructions.

### Quantitative real-time PCR

Total RNA was extracted from the sample using RNAiso Plus (TaKaRa, Dalian, China). Total RNA was subjected to reverse transcription using the PrimeScript RT reagent kit with gDNA Eraser (Perfect Real Time) (TaKaRa). Quantitative real-time PCR was performed using the Bio-Rad^®^ CFX96 PCR System (Bio-Rad, CA, USA), and the relative gene expression was normalized to β-actin as the internal control. The primer sequences of the target genes are described in Table 2.

**Table 2:** Real-time fluorescence quantitative PCR primer sequences.

**Gene**	**Primer (5′-3′)**		**Product size (bp)**
TH	F	CTCCCAGGACATTGGACTTGC	153
	R	TCTCCATAGGAAGACAGCAGCC	
α-syn	F	AAGAAGGACCAGATGGGCAAG	135
	R	GGCTTCAGGCTCATAGTCTTGG	
SOD1	F	TGGAGACCTGGGCAATGTGA	147
	R	CCACCTTTGCCCAAGTCATC	
CAT	F	GGTCACCGGCACATGAATGG	100
	R	CCTGGTCGGTCTTGTAATGGAAC	
GPX-1	F	CCAGGAGAATGGCAAGAATGA	138
	R	AGGAAGGTAAAGAGCGGGTGA	
TNF-α	F	CATTGCTGCCAACATCATCCA	92
	R	CCAGAGCGGCTACTCAGAAACT	
IL-4	F	GTTGCCTTCTTGGGACTGATGT	96
	R	TCTGTTGTGGGTGGTATCCTCTG	
IL-6	F	CTGTTGCTGCTACTGAACCTGG	134
	R	CGCTTTTGAGCTAAGGGAGTTG	
DAT	F	GGAGTGCTCATTGAAGCCATTG	116
	R	TTCCAGCATAGCCGCCAGTA	
β-actin	F	CATCCGTAAAGACCTCTATGCCAAC	171
	R	ATGGAGCCACCGATCCACA	

### Statistical analysis

The regions of the mouse brain were located according to an anatomical map by Pingyu Wang [71]. A one-way ANOVA with LSD correction was used to compare different groups. Data are expressed as the mean ± standard deviation (X±SD) for bodyweight, food uptake, behavioral test and staining, while the quantifications for iron concentration, qRT-PCR, and oxidative damage are presented as the mean ± SEM (X ± SEM). Analyses were performed using SPSS 20.0 software (IBM Corp, USA) for Windows, with the level of significance set at 0.05.

## Supplementary Material

Supplementary Figure 1
