# Supplementary material for: Iron overload resulting from the chronic oral administration of ferric citrate induces parkinsonism phenotypes in middle-aged mice
Source: Aging (Albany NY). 2019 Nov 7;11(21):9846–61. doi: 10.18632/aging.102433 (PMC6874424; doi:10.18632/aging.102433)
Supplement: Supplementary Figure 1 [file aging-11-102433-s001.pdf]

SUPPLEMENTARY FIGURE

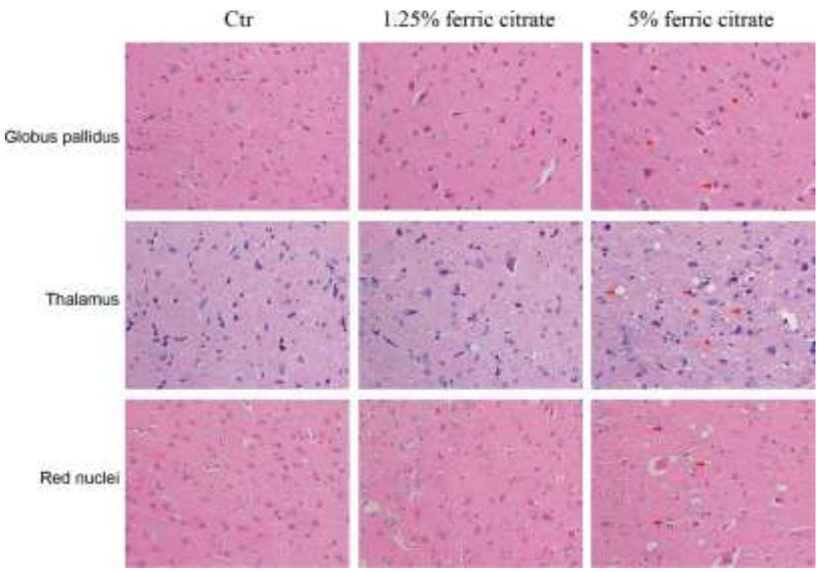

Supplementary Figure 1. Representative images of H&E staining display the histopathological damage in globus pallidus, thalamic and red nuclei of the brain induced by ferric citrate supplementation. Red Stars show white matter edema, red arrows show display nerve cell swelling.
